# Supplementary figures and images for: Global and local ancestry estimation in a captive baboon colony
Source: PLoS One. 2024 Jul 3;19(7):e0305157. doi: 10.1371/journal.pone.0305157 (PMC11221750; doi:10.1371/journal.pone.0305157)

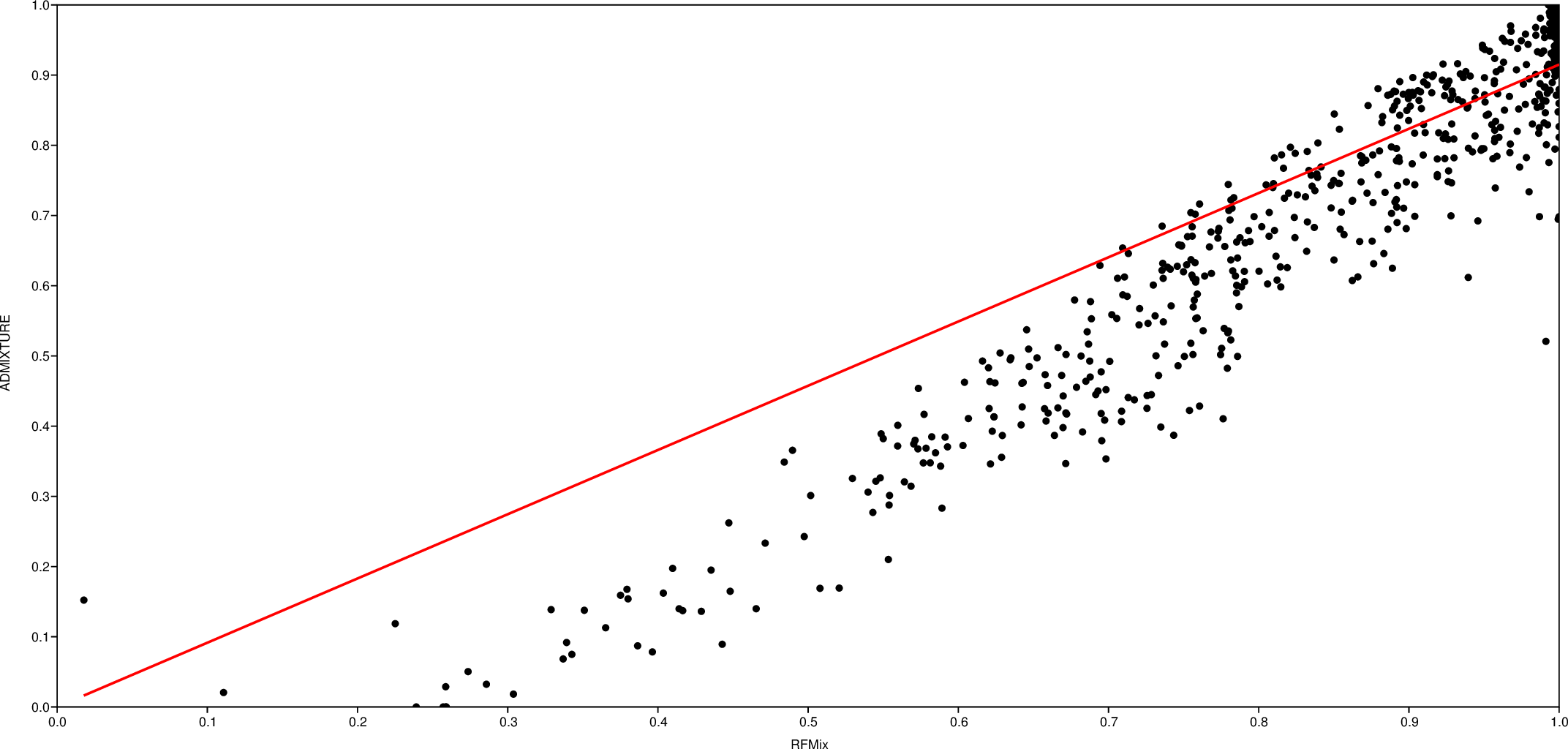

Supplement: S1 Fig — Global olive ancestry estimates from both ADMIXTURE [43] and RFMix [40] using the Beagle [38, 39] pipeline. There is excellent agreement between the two software with R2 = 0.9189. RFMix [40] appears to overestimate olive ancestry relative to ADMIXTURE [43]. Plot was generated in PAST v4.03 [60]. (TIF) [file pone.0305157.s001.tif]

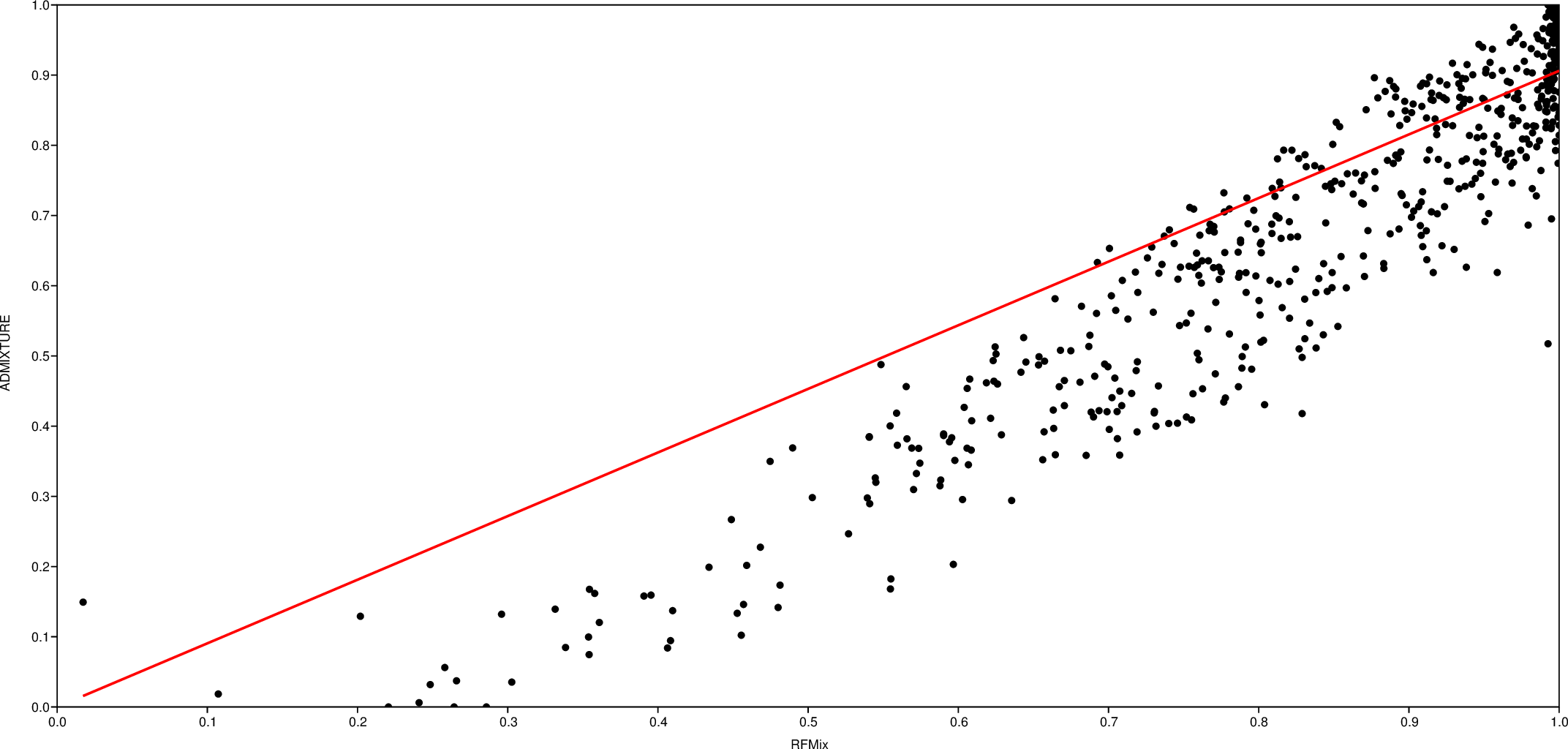

Supplement: S2 Fig — Global olive ancestry estimates from both ADMIXTURE [43] and RFMix [40] using the SHAPEIT5/IMPUTE5 [27, 41] pipeline. There is a slight drop in the congruence between the ancestry estimates compared to the Beagle pipeline with R2 = 0.8876. As seen in S1 Fig, RFMix [40] overestimates olive ancestry compared to ADMIXTURE [43]. Plot was generated using PAST [60]. (TIF) [file pone.0305157.s002.tif]

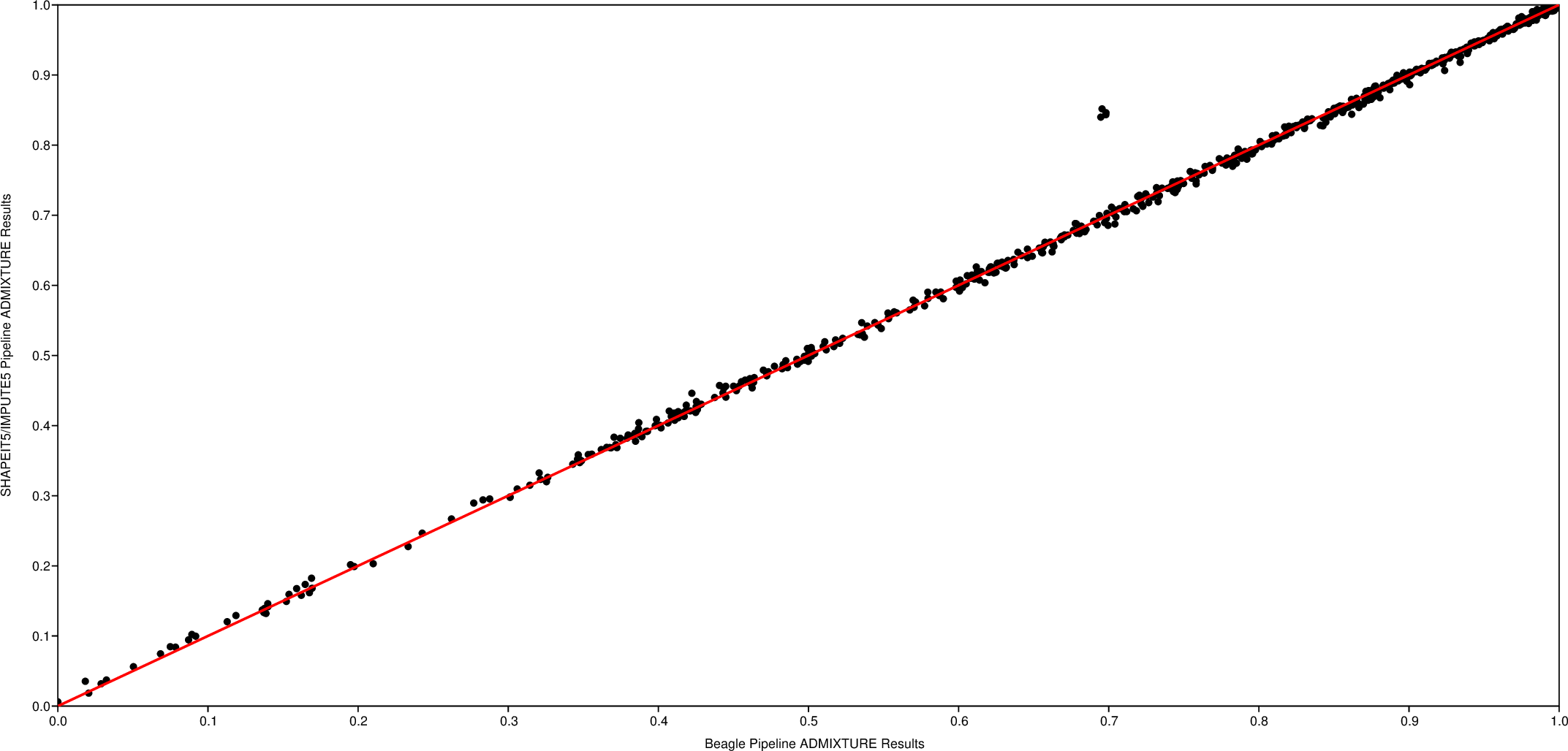

Supplement: S3 Fig — Global olive ancestry estimates using ADMIXTURE [43] from both pipelines. The software has an extremely strong association with R2 = 0.9982. Plot was generated in PAST [60]. (TIF) [file pone.0305157.s003.tif]

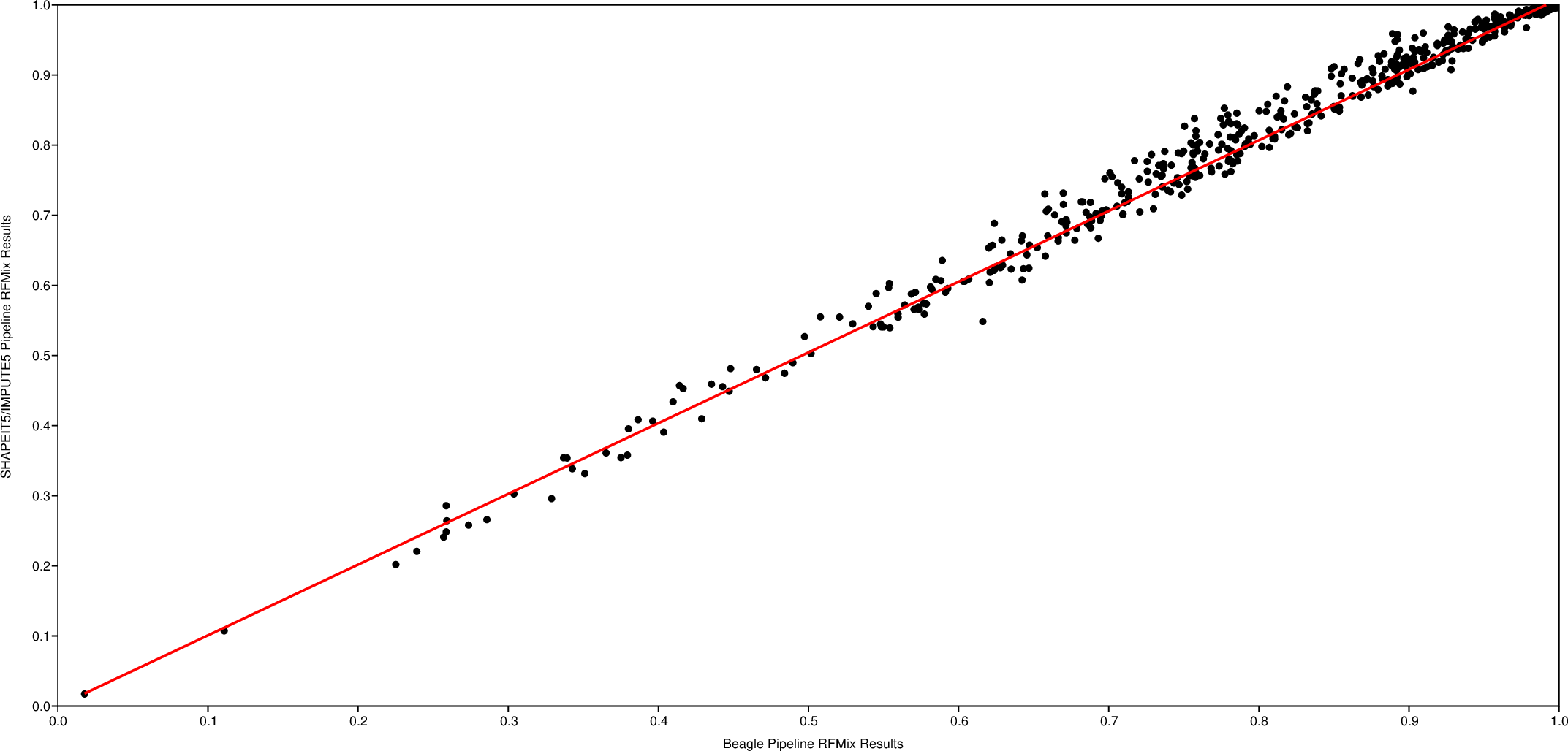

Supplement: S4 Fig — Global olive ancestry estimates using RFMix [40] from both pipelines. The software has a strong association with R2 = 0.9897 but shows less consistency in ancestry assignment than ADMIXTURE [43] when comparing both pipelines (S3 Fig). Plot was generated using PAST [60]. (TIF) [file pone.0305157.s004.tif]

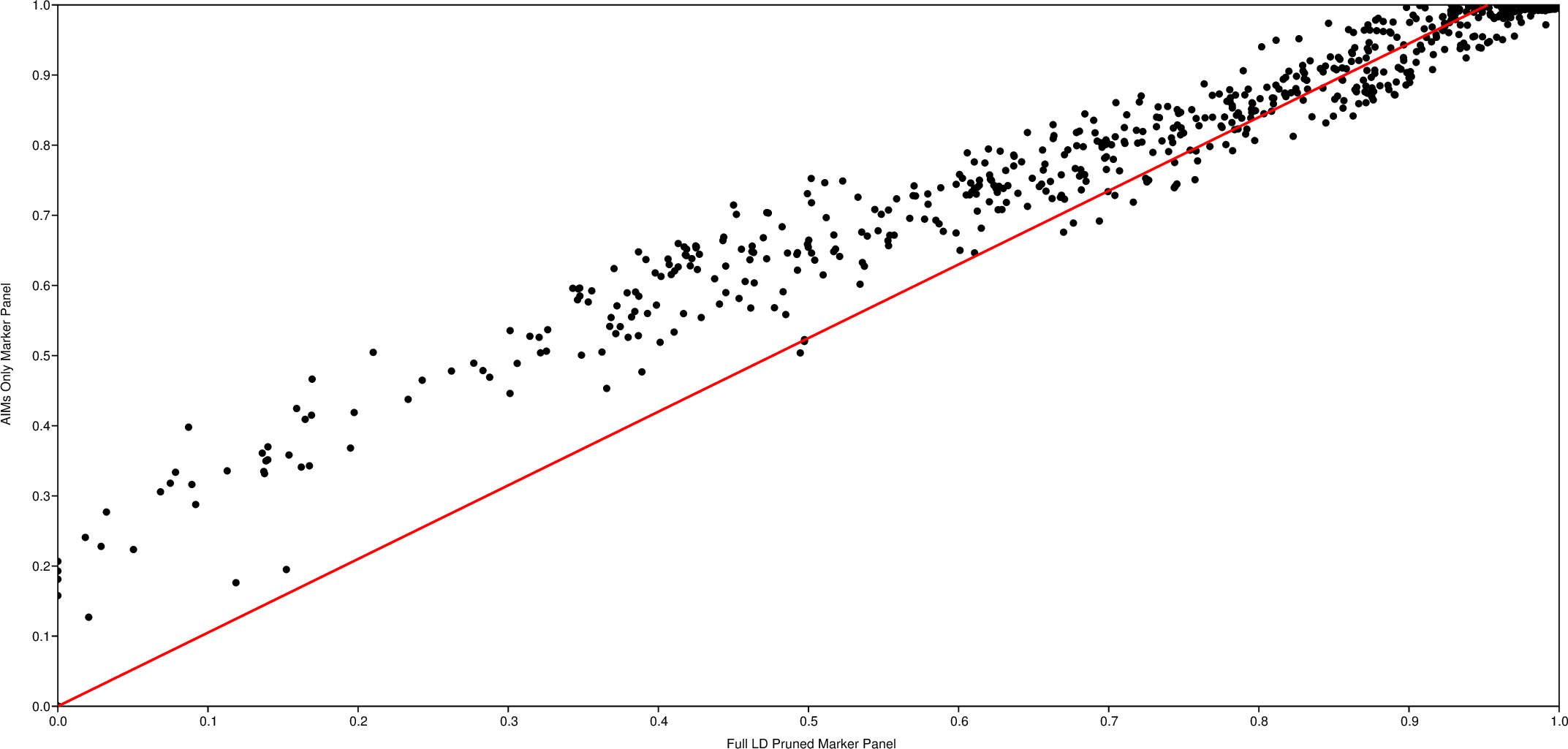

Supplement: S5 Fig — Global olive ancestry estimates from ADMIXTURE [43] using the Beagle pipeline [38, 39] LD pruned full marker set (n = 354,064) vs. just the AIMs (n = 1,747). The association is very strong (R2 = 0.9548), however, the AIMs overestimate olive ancestry, especially at low levels of ancestry compared to the full marker set. Plot was generated in PAST [60]. (TIF) [file pone.0305157.s005.tif]

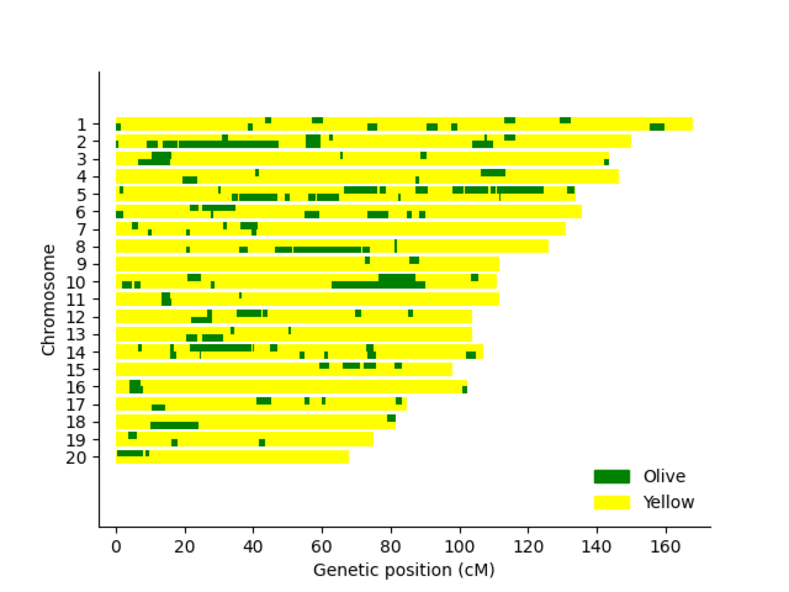

Supplement: S6 Fig — Sample 1X0102 is a purebred yellow founder (S2 Table). His local ancestry estimation based on RFMix [40] that has been corrected for phase switching using Tractor [46] showcases some historic olive ancestry. Plot was generated using haptools [49]. (TIF) [file pone.0305157.s006.tif]

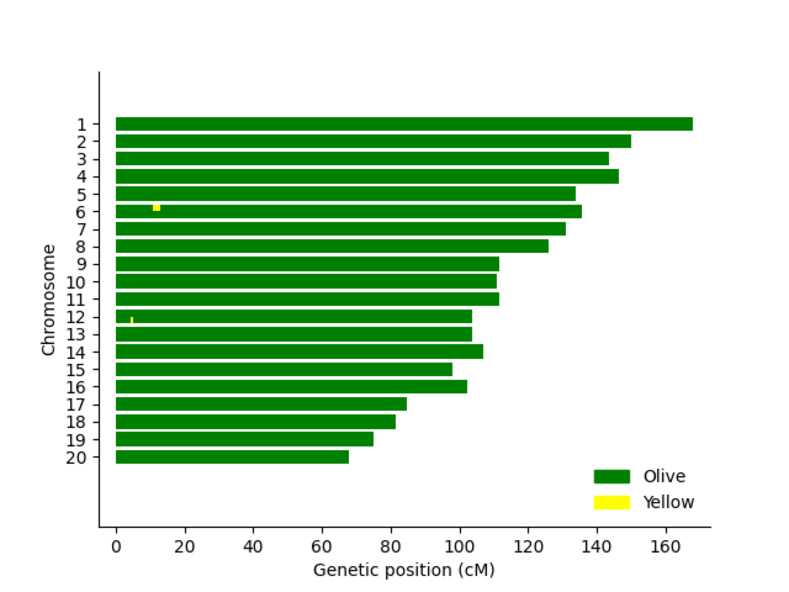

Supplement: S7 Fig — Sample 1X0026 is a purebred olive founder (S2 Table). Her local ancestry estimation that was estimated with RFMix [40] and corrected for phase switches using Tractor [46] largely agrees with the ADMIXTURE [43] global ancestry estimates. Plot was generated using haptools [49]. (TIF) [file pone.0305157.s007.tif]

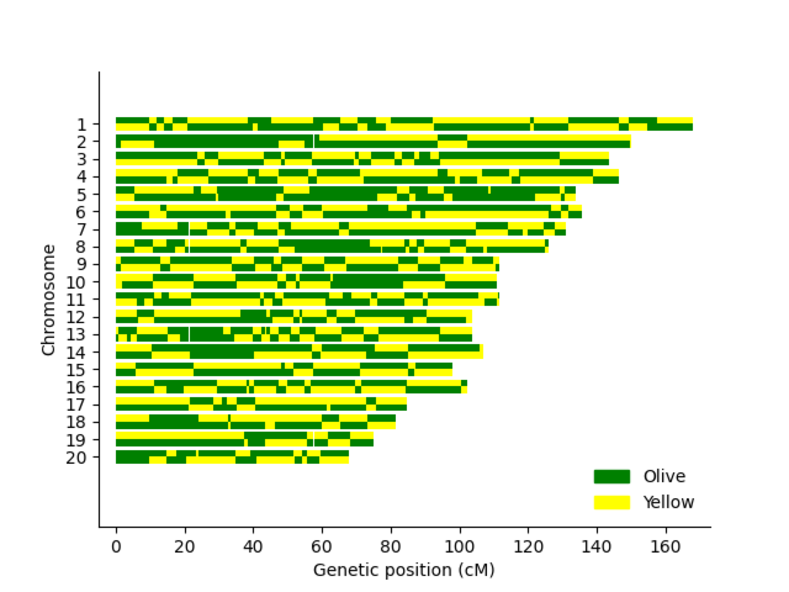

Supplement: S8 Fig — Sample 1X3837 is a first-generation hybrid offspring of a purebred olive (1X0026) mother and purebred yellow (1X0102) father. This plot, generated by haptools [49] shows the original output by Beagle [38, 39]. (TIF) [file pone.0305157.s008.tif]

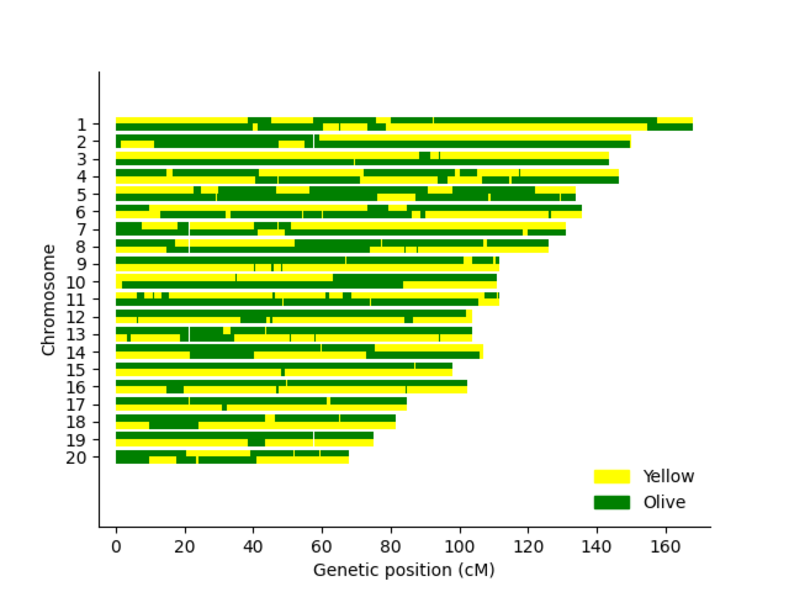

Supplement: S9 Fig — The same original Beagle [38, 39] data as S8 Fig but unkinked using Tractor [46]. Plot generated by haptools [49]. (TIF) [file pone.0305157.s009.tif]

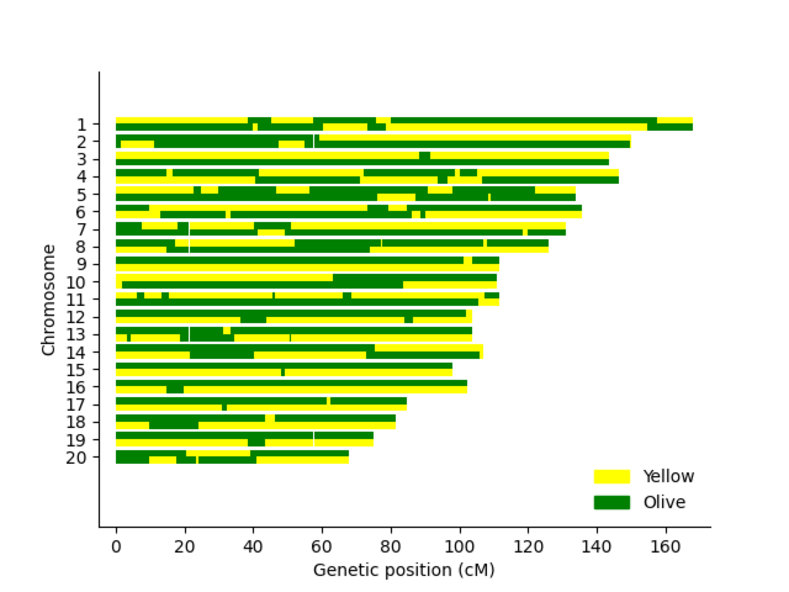

Supplement: S10 Fig — Beagle [38, 39] data with a second RFMix [40] round using the unkinked data from S9 Fig as input. The plot was generated using haptools [49]. (TIF) [file pone.0305157.s010.tif]

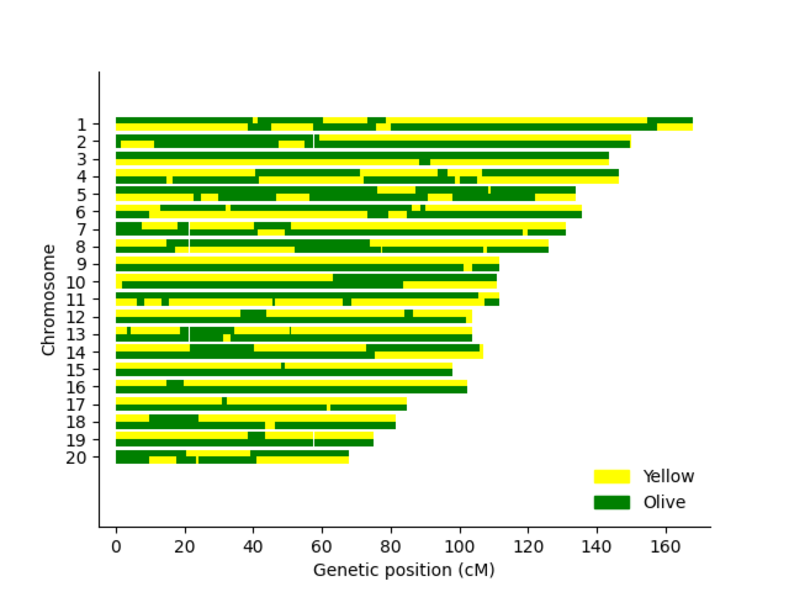

Supplement: S11 Fig — Beagle [38, 39] data with a second RFMix [40] round using the unkinked data from S9 as input followed by a second round of unkinking using Tractor [46]. Her local ancestry estimation done by RFMix [40] and corrected for phase switch errors using Tractor [46] still shows evidence of switch errors. Additionally, many of these switches do not align with what is seen in the parental karyograms (S6 and S7 Figs). Plot created using haptools [49]. (TIF) [file pone.0305157.s011.tif]

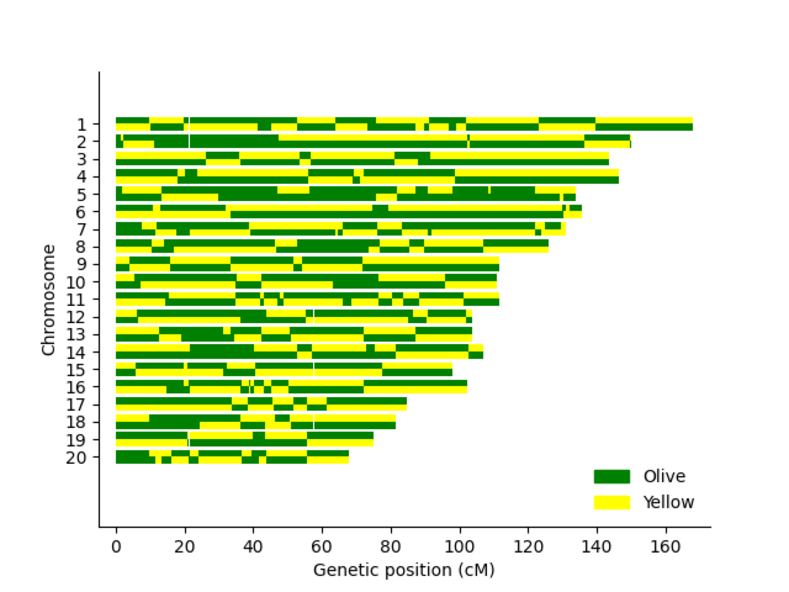

Supplement: S12 Fig — This plot, generated by haptools [49], shows the original output by SHAPEIT5/IMPUTE5 [27, 41]. (TIF) [file pone.0305157.s012.tif]

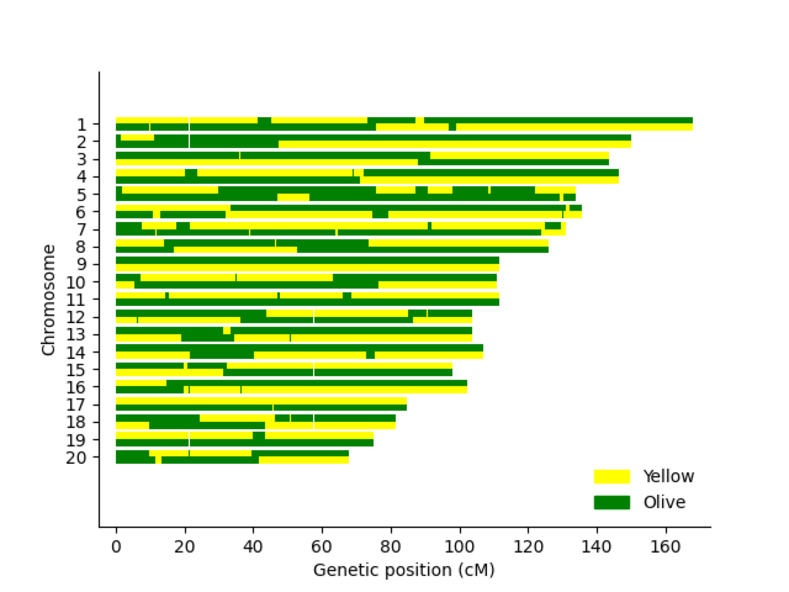

Supplement: S13 Fig — The same original SHAPEIT5/IMPUTE5 [27, 41] data as S12 Fig but unkinked using Tractor [46]. Plot generated by haptools [49]. (TIF) [file pone.0305157.s013.tif]

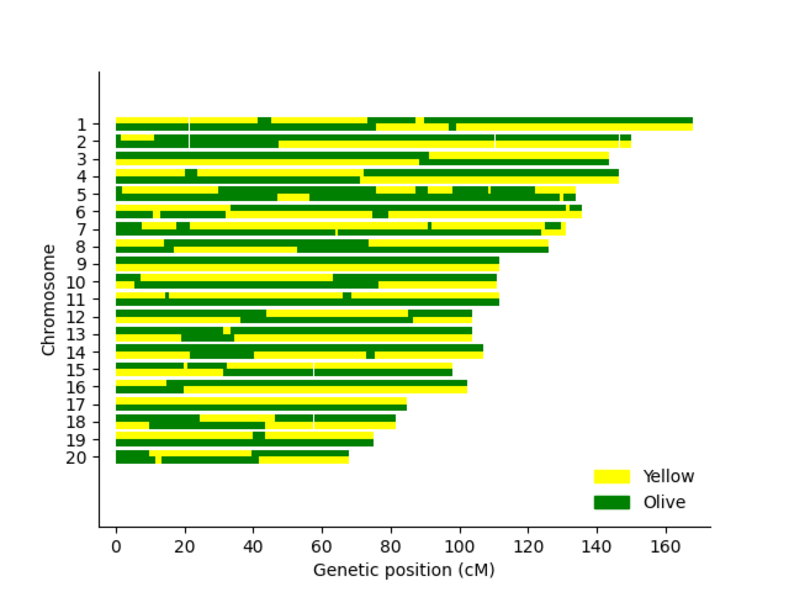

Supplement: S14 Fig — SHAPEIT5/IMPUTE5 [27, 41] data with a second RFMix [40] round using the unkinked data from S13 Fig as input. The plot was generated using haptools [49]. (TIF) [file pone.0305157.s014.tif]

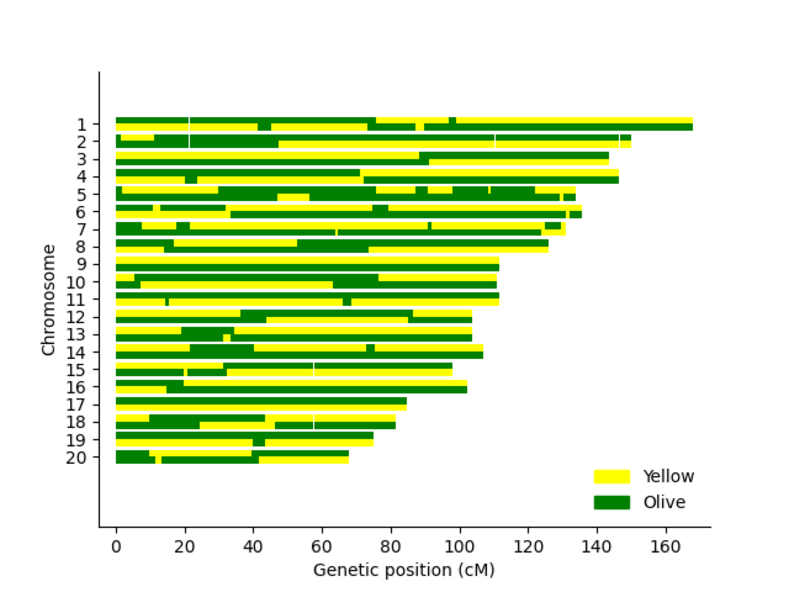

Supplement: S15 Fig — SHAPEIT5/IMPUTE5 [27, 41] data with a second RFMix [40] round using the unkinked data from S13 Fig as input followed by a second round of unkinking using Tractor [46]. While some switch errors and ancestry misassignments are still persistent, these are reduced relative to S11 Fig. Plot created using haptools [49]. (TIF) [file pone.0305157.s015.tif]

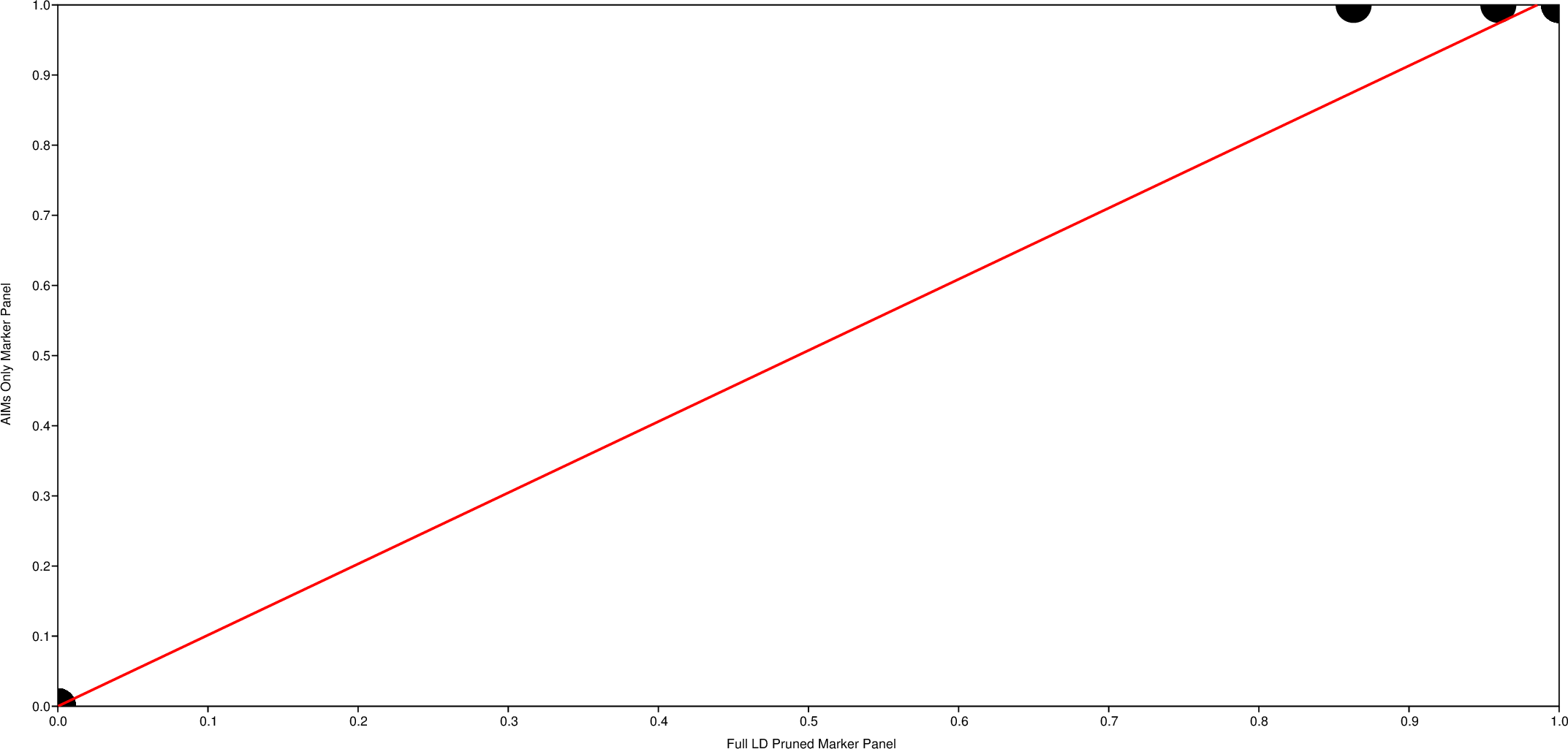

Supplement: S16 Fig — Global olive ancestry estimates from ADMIXTURE [43] using an LD pruned marker panel (n = 334,469 for Vilgalys et al. [20] samples; n = 152,878 for Rogers et al. [2] samples) vs. just our panel of AIMs (n = 1,747). The association is extremely strong (R2 = 0.99723). The AIMs still tend to overestimate the amount of olive ancestry even in these wild populations. Plot was generated in PAST [60]. (TIF) [file pone.0305157.s016.tif]
